# Tumor-cell PD-L1 expression, mismatch repair status, and survival outcomes in colorectal carcinoma: a retrospective Pakistani cohort study

**DOI:** 10.3389/fonc.2026.1939073

**Published:** 2026-09-14

**Authors:** Kanza Atif, Aiman Ajmeer, Abdal Ahmad, Fozia Rauf, Umer Khan, Sarah Yousuf, Bilal Ahmad, Sadia Qazi

**Affiliations:** 1 College of Medicine, Alfaisal University, Riyadh, Saudi Arabia; 2 Department of Pathology, Peshawar Medical College, Riphah International University, Peshawar, Pakistan; 3 Department of Medicine, Peshawar Medical College, Riphah International University, Peshawar, Pakistan; 4 Department of Oncology, Peshawar Medical College, Riphah International University, Peshawar, Pakistan; 5 Department of Anatomy, College of Medicine, Alfaisal University, Riyadh, Saudi Arabia

**Keywords:** colorectal carcinoma, immunohistochemistry, microsatellite instability, mismatch repair, Pakistan, programmed death-ligand 1, survival

## Abstract

**Background:**

Tumor-cell programmed death-ligand 1 (PD-L1) expression has been investigated as a biomarker for colorectal carcinoma (CRC), but the reported positivity rates vary by antibody clone, scoring method, and threshold. Tumor-cell PD-L1 is not a validated companion diagnostic tool for CRC. We assessed whether PD-L1 expression scored with the ZR3 clone functions as a clinicopathological or prognostic biomarker in a Pakistani cohort, using microsatellite instability/mismatch-repair (MSI/MMR) status as the reference biomarker.

**Methods:**

We retrospectively evaluated 60 patients with CRC diagnosed between 2020 and 2023. PD-L1 expression was assessed using the tumor proportion score (TPS) and ZR3 antibody. MSI was determined using polymerase chain reaction-based fragment analysis, and MMR status was determined using four-protein immunohistochemistry. Associations between binary PD-L1 status and 10 clinicopathological variables were analyzed and adjusted using the Benjamini–Hochberg false discovery rate procedure. Overall survival (OS; n=60), disease-free survival (DFS; n=44), and progression-free survival (PFS; n=16) were the exploratory outcomes.

**Results:**

At a TPS threshold of ≥1%, PD-L1 positivity was identified in 43 of 60 tumors (71.7%), including 31 low-positive and 12 high-positive tumors. This rate exceeded that of most CRC TPS series and likely reflected clone- and threshold-specific assay behavior. No clinicopathological variables were associated with binary PD-L1 status after multiplicity correction. MSI-H/dMMR was present in 12 cases (20.0%) and was associated with the three-level PD-L1 category (Fisher–Freeman–Halton p=0.0017; Cramer’s V = 0.485), but not with binary positivity (p=0.151); the association was confined to the high-positive group. PD-L1 status was not associated with OS (HR 1.43, 95% CI 0.40–5.05; p=0.580), DFS (HR 0.46, 95% CI 0.15–1.45; p=0.185), or PFS (HR 0.67, 95% CI 0.20–2.20; p=0.505). Older age, rather than PD-L1 status, was associated with mortality (adjusted HR 1.68 per 10 years, 95% CI 1.09–2.57; p=0.018).

**Conclusions:**

ZR3-based tumor-cell PD-L1 TPS was not associated with clinicopathological characteristics or survival in this cohort. The high positivity rate should be interpreted as assay-specific, while the enrichment of MSI-H/dMMR tumors in the high-positive tier was consistent with established biology. ZR3-based TPS should remain a research measure and should not independently guide immunotherapy selection; MSI/MMR testing remains the clinically actionable biomarker for CRC.

## Introduction

Colorectal cancer (CRC) is the third most commonly diagnosed malignancy and the second leading cause of cancer-related death worldwide. GLOBOCAN 2022 estimated approximately 1.9 million new cases and 904,000 deaths, with the burden projected to rise by 2040 owing to population growth, aging, and lifestyle changes (1, 2). In Pakistan, the true burden is hard to quantify because national registry coverage is incomplete, and evidence is derived largely from regional registries, hospital series, and pooled estimates (3–6), while limited screening, low awareness, and constrained diagnostic access contribute to delayed, advanced-stage presentation (7).

Prognostic assessment of CRC still relies on established clinicopathological features, including histological subtype and grade, depth of invasion, nodal involvement, vascular and perineural invasion, margin status, and pathological stage (8–10). However, patients with similar morphology and stage may differ in recurrence, treatment response, and survival, motivating interest in molecular and immune biomarkers that complement conventional assessments.

The programmed death-1/programmed death-ligand 1 (PD-1/PD-L1) pathway is a major immune checkpoint mechanism of tumor immune escape: PD-1 on activated lymphocytes binds PD-L1 on tumor and immune cells, suppressing T-cell activation, cytokine production, and cytotoxicity, and weakening antitumor immunity (11–13). In CRC, immune checkpoint inhibition chiefly benefits tumors with microsatellite instability-high or deficient mismatch repair (MSI-H/dMMR), where pembrolizumab and nivolumab show durable, clinically meaningful activity (14–17). Therefore, MSI/MMR status is an established, clinically actionable biomarker for immunotherapy selection, whereas tumor-cell PD-L1 expression alone is not a validated companion biomarker for this indication (18).

Tumor-cell PD-L1 has been widely studied as a prognostic and predictive biomarker in CRC. Reported positivity rates vary considerably with the antibody clone, staining platform, tissue processing, scoring system, evaluated compartment, and threshold (19, 20), and the prognostic evidence is similarly inconsistent: meta-analyses link higher PD-L1 to adverse features or survival in some groups, but heterogeneity and conflicting cohorts prevent firm conclusions (21, 22). South Asian studies are also variable; Srivastava et al. related PD-L1 to clinicopathological variables, microsatellite instability (MSI) status, and BRAF mutation, and Katta et al. related it to poor differentiation, tumor-infiltrating lymphocytes, and higher T and N categories (23, 24). This indicates that its significance depends on the population, assay, scoring method, and molecular profile.

However, evidence from Pakistan remains limited. Ahmad et al. investigated the relationship among tumor-infiltrating lymphocytes, tumor stage, and PD-L1 expression in colorectal carcinoma and provided initial local evidence from a Pakistani population (25). Although that study addressed the association of PD-L1 with the immune microenvironment and tumor stage, the present investigation evaluated an independent and non-overlapping cohort and integrated tumor-cell PD-L1 tumor proportion score (TPS) with PCR-based MSI testing, four-protein MMR immunohistochemistry, and longitudinal clinical outcomes. The present study had a deliberately cautionary aim: to determine the frequency of tumor cell PD-L1 expression using the ZR3 clone and to test whether it behaves as a usable clinicopathological or prognostic biomarker in a Pakistani CRC cohort, with MSI/MMR status as the reference clinically actionable biomarker. Because tumor-cell PD-L1 is not a validated companion diagnostic for CRC, the analysis was designed to characterize assay behavior rather than to establish PD-L1 as a biomarker. Overall, disease-free and progression-free survival were examined only as prespecified secondary exploratory outcomes using endpoint-specific analysis populations.

## Materials and methods

### Study design and setting

This analytical retrospective cohort study was conducted at the Department of Pathology, Peshawar Medical College, Riphah International University, Peshawar, Pakistan. Formalin-fixed paraffin-embedded (FFPE) blocks from patients with histologically confirmed CRC diagnosed between January 2020 and July 2023 were retrieved and analyzed between August 2023 and September 2025, with follow-up updated through September 2025. The reporting followed the STROBE statement (26).

Each record represented a unique patient, and only one representative FFPE block was included for each patient. Duplicate resections, repeat specimens, and multiple blocks from the same patients were excluded from the analytical cohort.

### Participants, sample size, and eligibility

Of the 78 archived FFPE blocks screened, 18 were excluded (four not CRC, 10 with inadequate viable tumor or extensive necrosis, and four with incomplete records), leaving 60 unique patients, one representative block each (**Figure 1**). The sample size was based on a chi-square association (effect size 0.40, two-sided alpha 0.05, 80% power, two degrees of freedom), yielding approximately 60 cases; survival analyses were exploratory and not part of the power calculation.

**Figure 1:**
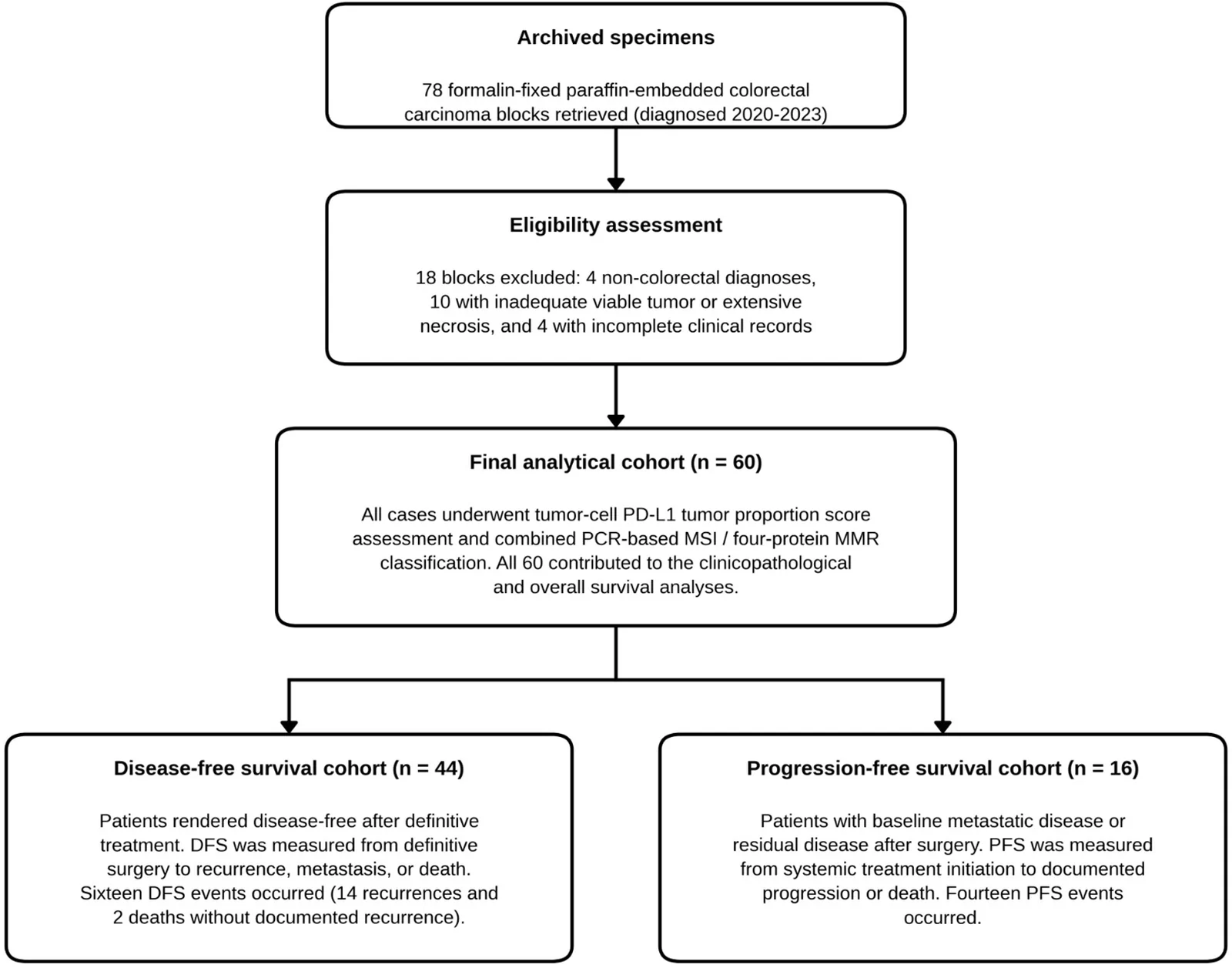
Participant flow and endpoint-specific analysis populations.

Eligible cases had histologically confirmed CRC, adequate fixation, viable tumors for immunohistochemistry, and complete clinical and pathological records. Post-treatment specimens with therapy-related changes that could compromise grading or PD-L1 interpretation were also excluded.

### Ethics

The Institutional Review Board of Prime Foundation Pakistan approved the study (Prime/IRB/2023-1015; August 31, 2023) and waived individual informed consent because only anonymized archival specimens and retrospective records were used in the study. Departmental permission was obtained before accessing the blocks and records. Data were de-identified before analysis, and the study was conducted in accordance with the Declaration of Helsinki principles.

### Histopathological assessment

FFPE sections were cut at 5 µm and stained with hematoxylin and eosin. Diagnosis, tumor site, histological subtype, differentiation, lymph node status, vascular invasion, perineural invasion, and pathological stage were reviewed. Classification and grading followed the 2019 World Health Organization classification of digestive system tumors (8), and staging followed the eighth edition of the American Joint Committee on Cancer TNM system (9). Representative images of each grade are presented in **Figure 2**. Well- and moderately differentiated tumors were grouped as low/moderate grade for selected secondary analyses, whereas poorly differentiated tumors were classified as high grade.

**Figure 2:**
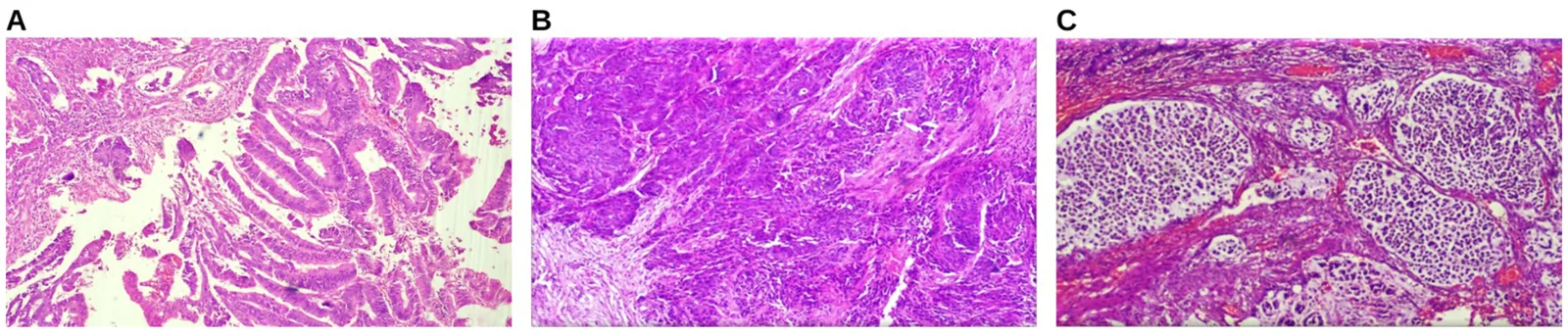
Histological grading of colorectal adenocarcinoma on hematoxylin and eosin sections (10× objective; ×100 total magnification). **(A)** Well-differentiated adenocarcinoma with well-formed glands. **(B)** Moderately differentiated adenocarcinoma with irregular and fused glands. **(C)** Poorly differentiated adenocarcinoma with minimal gland formation and marked atypia.

### PD-L1 immunohistochemistry and scoring

PD-L1 immunohistochemistry was performed on 5-micrometer FFPE sections using a ready-to-use prediluted ZR3 rabbit monoclonal antibody (Cell Marque, Rocklin, CA, United States) per the manufacturer’s instructions, with deparaffinization, graded rehydration, heat-induced epitope retrieval, primary antibody incubation, polymer-horseradish peroxidase detection (Cell Marque Universal Detection Kit), 3,3′-diaminobenzidine development, and hematoxylin counterstaining; tonsils served as the external positive control and primary antibody omission as the negative control, consistent with published PD-L1 reporting frameworks (27, 28). Because contemporaneous run sheets were not retained, platform-specific retrieval buffer, pH, incubation times, and catalog or lot numbers were not imputed from the secondary literature and are acknowledged as a retrospective-methods limitation.

Tumor-cell PD-L1 expression was scored as TPS, defined as the percentage of viable tumor cells with partial or complete membrane staining. Cytoplasmic-only staining, stromal staining, immune cell staining, and necrotic areas were excluded. At least 100 viable tumor cells were assessed per sample. Two pathologists independently reviewed each slide blinded to the clinical outcomes, and disagreements were resolved by consensus. Individual pre-consensus scores were not retained; consequently, the interobserver agreement could not be calculated. A TPS <1% was classified as negative, TPS 1-49% as low-positive, and TPS ≥ 50% as high-positive (19, 27–31). Each case was assigned directly to one of these three categories at the time of consensus scoring, and the underlying numerical percentage was not recorded in the study database; therefore, the analyses reported here treat the tumor proportion score as an ordered three-level variable throughout. Representative staining at each level, together with an external positive control, is shown in **Figure 3**. ZR3 is not an approved CRC companion-diagnostic assay; results therefore represent assay-specific laboratory data and are not interchangeable with validated companion-diagnostic platforms (30, 32).

**Figure 3:**
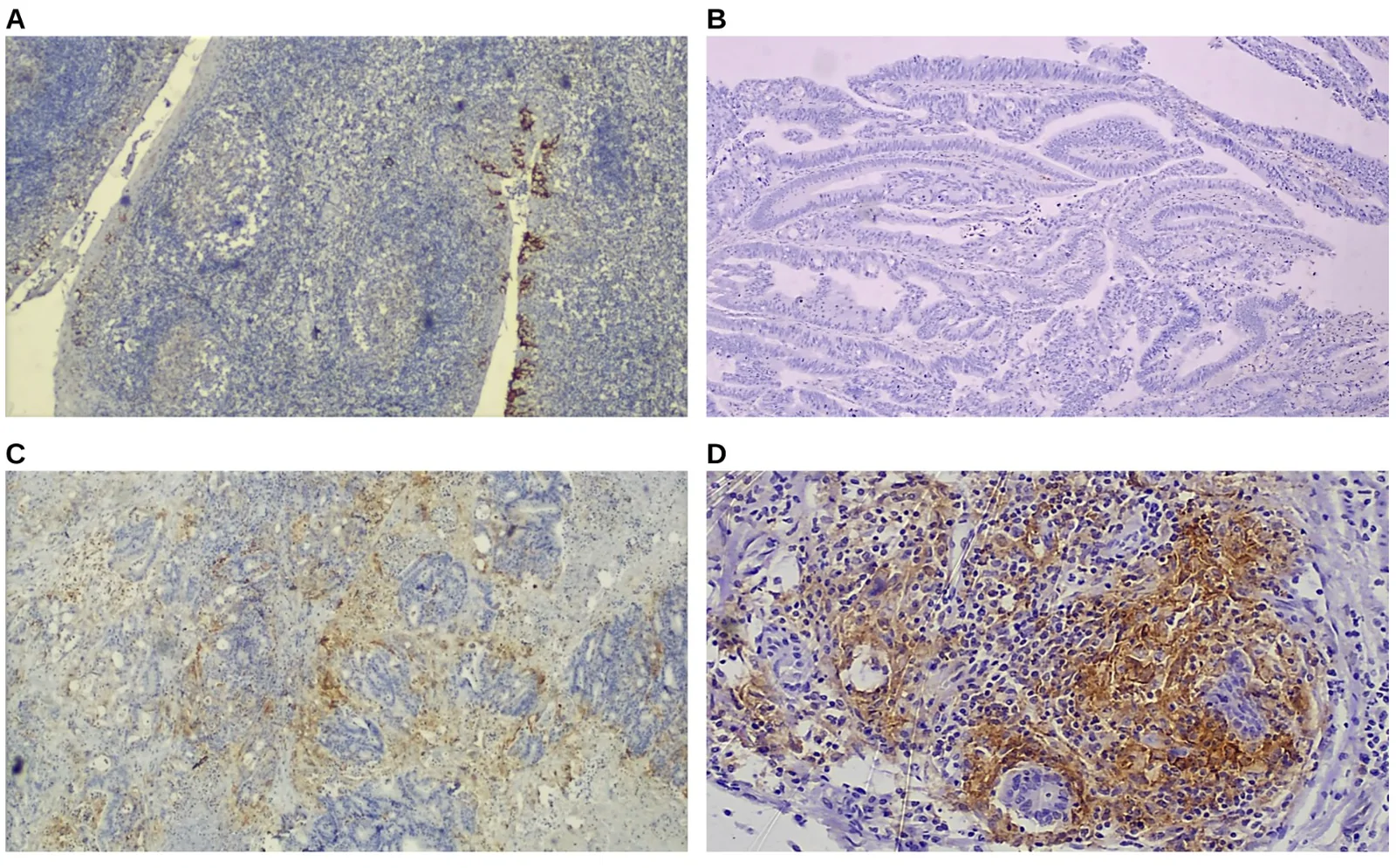
PD-L1 immunohistochemical staining using the ZR3 antibody (20× objective; ×200 total magnification). **(A)** Tonsil external positive control. **(B)** PD-L1-negative colorectal carcinoma (TPS <1%). **(C)** Low-positive carcinoma (TPS 1-49%). **(D)** High-positive carcinoma (TPS ≥50%). Only partial or complete membranous staining of viable tumor cells was counted towards the tumor proportion score; cytoplasmic, stromal, and immune-cell staining was excluded.

### MSI and MMR assessment

Microsatellite instability was assessed using the same formalin-fixed paraffin-embedded block used for immunohistochemistry. A hematoxylin and eosin-stained section was first reviewed by a pathologist, who marked a region of high neoplastic cell content, and consecutive unstained sections were cut from that region for tumor-enriched macrodissection so that the analyzed material was not diluted by non-neoplastic tissue. Genomic DNA was extracted from the macrodissected material, and its concentration and purity were confirmed prior to amplification.

MSI status was assessed by fluorescent multiplex (pentaplex) PCR-based fragment analysis using the quasimonomorphic mononucleotide markers BAT-25, BAT-26, NR-21, NR-22, and NR-24, as originally described by Suraweera et al. (33). This panel was used because these five mononucleotide repeats are quasimonomorphic in the general population, allowing MSI to be determined from tumor DNA alone without a matched non-neoplastic sample (33). This property was subsequently characterized across multiple populations for later implementation of the pentaplex, in which NR-27 replaced NR-22 (34). Current guidance recommends a five-marker PCR panel containing at least BAT-25 and BAT-26 as the molecular reference method for MSI testing (35). The five loci were co-amplified in a single multiplex (pentaplex) reaction using fluorescently labeled primers, so that all five markers were amplified from the same aliquot of tumor DNA, under identical conditions. The amplification products were then combined with an internal size standard and separated by capillary electrophoresis, and allele sizes were read from the resulting electropherograms, with the co-injected size standard providing the reference scale for fragment length. Each sample was run with its controls in the same batch. Each marker was scored independently by an observer blinded to the PD-L1 result as unstable when the tumor trace contained one or more novel alleles falling outside the quasimonomorphic size range established for that marker, that is, a shift in repeat length relative to the reference allele distribution; markers that failed to amplify were treated as non-informative and were not counted towards the marker tally. Classification followed the international criteria agreed at the National Cancer Institute workshop and retained in the revised Bethesda guidelines (36, 37): tumors showing instability at two or more loci were classified as microsatellite instability-high (MSI-H), tumors showing instability at one locus were classified as microsatellite instability-low (MSI-L), and tumors without instability at any of the five loci were classified as microsatellite stable (MSS). Positive and no-template controls were included in every run and were required to provide the expected profile before a run was accepted.

MMR protein expression was assessed by immunohistochemistry for MLH1, MSH2, MSH6, and PMS2, with nuclear staining in non-neoplastic epithelial, stromal, and lymphoid cells used as the internal control. Complete loss of tumor cell nuclear staining with retained internal control indicated loss of the corresponding protein; deficient MMR (dMMR) was defined as loss of at least one protein, and proficient MMR (pMMR) required retention of all four proteins (38, 39). Loss was defined when tumor-cell nuclei were entirely unstained in the presence of an unequivocally stained internal control. Sections in which the internal control was absent or equivocal were regarded as non-informative and repeated. Cases were classified as MSI-H/dMMR when PCR-based MSI and MMR immunohistochemistry were concordantly abnormal and MSS/pMMR when both were normal; no MSI-low, equivocal, or discordant cases were observed.

### Treatment classification and outcome definitions

Treatment was categorized by clinical intent (curative or palliative) and recorded treatment combination. The generic term treatment received is used because chemotherapy, radiotherapy, targeted therapy, and immunotherapy were delivered in different perioperative or metastatic settings; they were not all adjuvant treatments.

OS was measured from the baseline diagnosis to death from any cause, censoring living patients at the last follow-up. DFS, restricted to the 44 patients who were rendered disease-free after definitive treatment, was defined as the time from definitive surgery to the first locoregional recurrence, distant metastasis, or death. PFS, in the 16 patients with baseline metastatic or residual disease, ranged from systemic treatment initiation to progression or death, with event-free patients censored at the last follow-up. This endpoint-specific classification prevented patients with persistent or metastatic disease from being analyzed as disease-free at the baseline.

### Statistical analysis

The patient-level dataset was analyzed in Python 3.13 (SciPy 1.17, statsmodels 0.14.6, lifelines 0.30.0); exact tests for r × 2 contingency tables were computed with the network algorithm implemented in R 4.5.3. Continuous variables are summarized as medians and interquartile ranges (IQR) and were compared using the Mann-Whitney U test. Categorical variables are presented as n (%). Fisher’s exact test was used for 2 × 2 tables, and the Fisher-Freeman-Halton exact test for sparse 3 × 2 tables. Effect sizes were reported as Phi, Cramér’s V, or Cliff’s delta, as appropriate. All tests were two-sided, and p<0.05 was considered significant.

The primary analysis evaluated binary PD-L1 status against 10 baseline clinicopathological variables: continuous age, sex, histological subtype, histological grade, tumor site, pathological stage, lymph node status, vascular invasion, perineural invasion, and MSI/MMR status. Age group and treatment variables were descriptive and were excluded from this multiplicity family to avoid duplicate or post-baseline testing. Benjamini-Hochberg correction controlled the false discovery rate across these 10 comparisons, and adjusted q-values are reported with raw p-values; q<0.05 was required for significance in this family.

Survival was estimated using Kaplan-Meier methods with log-rank tests, and Cox proportional hazards models provided HRs with 95% CIs. The OS model included age per 10-year increase and binary PD-L1 status (17 deaths and two covariates). Because the histological grade was non-proportional, the adjusted DFS estimate was obtained from a grade-stratified Cox model, and PFS was analyzed univariably, given that only 16 patients (four PD-L1-negative) were included. Proportional hazards assumptions were checked using Schoenfeld residuals, and median follow-up was estimated using reverse Kaplan-Meier.

Sensitivity analyses were performed to examine whether the ≥1% dichotomy obscured the information carried by the staining level. All analyses were repeated with PD-L1 entered as a three-level category (negative, low-positive, high-positive): the same family of 10 baseline variables with Benjamini-Hochberg correction, linear-by-linear trend tests across the ordered tiers, Kaplan-Meier estimation with global log-rank tests, Cox models entering the tier both as a single ordinal term scored 0/1/2 and as separate indicator variables with the negative tier as reference, and logistic regression models with MSI-H/dMMR as the outcome for each PD-L1 parameterization. Because several cells in these logistic models were sparse, their confidence intervals were profile-likelihood intervals, and their p-values were likelihood ratio tests. These analyses were secondary to the binary primary analysis and were interpreted as exploratory; they are reported in full in the **Supplementary Material**.

## Results

### Cohort characteristics and PD-L1 frequency

The final cohort included 60 patients with CRC. The median age was 57.5 years (IQR 40.5-69.3), 49 patients (81.7%) were male, and 46 (76.7%) had stage III or IV disease. PD-L1 TPS was negative in 17 cases (28.3%), low-positive in 31 (51.7%), and high-positive in 12 (20.0%); therefore, the overall positivity was 71.7%. MSI-H/dMMR was identified in 12 (20.0%) and MSS/pMMR in 48 (80.0%) cases. Forty-eight patients were treated with curative intent, and 12 were treated with palliative intent (**Tables 1**, **2**; **Figure 1**; **Supplementary Table S1**).

**Table 1:** Baseline clinicopathological characteristics according to binary PD-L1 status.

Variable	Category	Overall (N = 60)	PD-L1 negative (n=17)	PD-L1 positive (n=43)	p	q (BH)
Age, years	Median (IQR)	57.5 (40.5-69.3)	63.0 (51.0-68.0)	53.0 (39.0-69.5)	0.475	0.594
Sex	Female	11 (18.3%)	5 (29.4%)	6 (14.0%)	0.265	0.529
	Male	49 (81.7%)	12 (70.6%)	37 (86.0%)		
Histological subtype	Adenocarcinoma	52 (86.7%)	16 (94.1%)	36 (83.7%)	0.420	0.594
	Mucinous adenocarcinoma	8 (13.3%)	1 (5.9%)	7 (16.3%)		
Histological grade	Well differentiated	13 (21.7%)	3 (17.6%)	10 (23.3%)	0.376	0.594
	Moderately differentiated	24 (40.0%)	5 (29.4%)	19 (44.2%)		
	Poorly differentiated	23 (38.3%)	9 (52.9%)	14 (32.6%)		
Tumor site	Left-sided	40 (66.7%)	14 (82.4%)	26 (60.5%)	0.136	0.433
	Right-sided	20 (33.3%)	3 (17.6%)	17 (39.5%)		
Pathological stage	II	14 (23.3%)	7 (41.2%)	7 (16.3%)	0.173	0.433
	III	32 (53.3%)	7 (41.2%)	25 (58.1%)		
	IV	14 (23.3%)	3 (17.6%)	11 (25.6%)		
Lymph node status	pN0	22 (36.7%)	9 (52.9%)	13 (30.2%)	0.139	0.433
	pN1-pN2	38 (63.3%)	8 (47.1%)	30 (69.8%)		
Vascular invasion	Absent	31 (51.7%)	8 (47.1%)	23 (53.5%)	0.777	0.777
	Present	29 (48.3%)	9 (52.9%)	20 (46.5%)		
Perineural invasion	Absent	48 (80.0%)	13 (76.5%)	35 (81.4%)	0.726	0.777
	Present	12 (20.0%)	4 (23.5%)	8 (18.6%)		
MSI/MMR status	MSS/pMMR	48 (80.0%)	16 (94.1%)	32 (74.4%)	0.151	0.433
	MSI-H/dMMR	12 (20.0%)	1 (5.9%)	11 (25.6%)		

Values are n (%) unless otherwise stated. p-values: Mann-Whitney U for age, Fisher exact for 2 x 2 tables, and Fisher-Freeman-Halton exact for 3 x 2 tables. Benjamini-Hochberg q-values were calculated across 10 baseline variables. Effect sizes were small to modest (absolute Cliff’s delta 0.120; Phi 0.055-0.222; Cramér’s V 0.190-0.265).

**Table 2:** Treatment and endpoint-specific analysis populations.

Variable	Category	Overall (N = 60)	PD-L1 negative (n=17)	PD-L1 positive (n=43)
Treatment intent	Curative	48 (80.0%)	14 (82.4%)	34 (79.1%)
	Palliative	12 (20.0%)	3 (17.6%)	9 (20.9%)
Treatment received	Chemotherapy	43 (71.7%)	11 (64.7%)	32 (74.4%)
	Chemotherapy + Radiotherapy	7 (11.7%)	5 (29.4%)	2 (4.7%)
	Chemotherapy + Targeted therapy	7 (11.7%)	1 (5.9%)	6 (14.0%)
	Chemotherapy + Immunotherapy	3 (5.0%)	0 (0.0%)	3 (7.0%)
Analysis population	Overall survival	60 (100.0%)	17 (100.0%)	43 (100.0%)
	Disease-free survival	44 (73.3%)	13 (76.5%)	31 (72.1%)
	Progression-free survival	16 (26.7%)	4 (23.5%)	12 (27.9%)

Percentages in endpoint rows use the full cohort as the denominator; group-specific counts identify the participants included in each survival analysis. Treatment categories describe recorded treatment combinations and should not all be interpreted as adjuvant therapy.

### Clinicopathological associations with binary PD-L1 status

No baseline clinicopathological variables were significantly associated with the binary PD-L1 status. The median age was 63.0 years in the PD-L1-negative group and 53.0 years in the positive group (p=0.475; q=0.594). Poor differentiation was present in 9/17 PD-L1-negative cases (52.9%) and 14/43 positive cases (32.6%); the three-level grade distribution was not associated with PD-L1 status (Fisher-Freeman-Halton p=0.376; q=0.594; Cramér’s V = 0.190). Tumor site, stage, lymph node status, vascular invasion, perineural invasion, histological subtype, sex, and MSI/MMR status also did not meet either the nominal or FDR-adjusted threshold (**Table 1**).

### MSI/MMR phenotype and PD-L1 expression

The three-level PD-L1 expression category was significantly associated with the MSI/MMR phenotype (Fisher-Freeman-Halton p=0.0017; Cramér’s V = 0.485). High-positive PD-L1 expression occurred in 7/12 MSI-H/dMMR tumors (58.3%) compared with 5/48 MSS/pMMR tumors (10.4%). In contrast, binary PD-L1 positivity was 91.7% in MSI-H/dMMR and 66.7% in MSS/pMMR tumors, which was not significant (Fisher p=0.151; Phi=0.222). MSI-H/dMMR was more frequent in right-sided tumors than in left-sided tumors (8/20, 40.0% vs. 4/40, 10.0%; p=0.014; Cramér’s V = 0.354) but was not associated with tumor grade or stage (**Table 3**). The dMMR patterns were paired MLH1/PMS2 loss in six cases, paired MSH2/MSH6 loss in three, isolated MSH6 loss in two, and isolated PMS2 loss in one case (**Supplementary Table S1**).

**Table 3:** MSI/MMR phenotype in relation to PD-L1 expression category and tumor site.

Variable	Category	MSS/pMMR (n=48)	MSI-H/dMMR (n=12)	Exact p	Effect size
PD-L1 expression category	Negative	16 (33.3%)	1 (8.3%)	0.0017	Cramér’s V = 0.485
	Low positive	27 (56.2%)	4 (33.3%)		
	High positive	5 (10.4%)	7 (58.3%)		
Tumor site	Left-sided	36 (75.0%)	4 (33.3%)	0.014	Cramér’s V = 0.354
	Right-sided	12 (25.0%)	8 (66.7%)		

The PD-L1 category comparison used the Fisher-Freeman-Halton exact test; tumor site used Fisher exact testing. Binary PD-L1 positivity was not significantly associated with MSI/MMR status (p=0.151).

### Overall survival

Seventeen deaths occurred during the follow-up period. Reverse Kaplan-Meier median follow-up for OS was 32.8 months, and median OS for the full cohort was 50.8 months. The survival outcomes of PD-L1-positive and PD-L1-negative patients did not differ (p=0.580). The univariable HR for PD-L1 positivity was 1.43 (95% CI 0.40-5.05; p=0.580). In the exploratory two-covariate model, each 10-year increase in age was associated with higher mortality (adjusted HR 1.68, 95% CI 1.09-2.57; p=0.018), whereas PD-L1 status was not (adjusted HR 2.00, 95% CI 0.55-7.29; p=0.293) (**Table 4**; **Figures 4**, **5**).

**Table 4:** Time-to-event outcomes according to binary PD-L1 status.

Endpoint	Cohort N/events	PD-L1 negative N/events; median months (95% CI)	PD-L1 positive N/events; median months (95% CI)	Log-rank p	Unadjusted HR (95% CI); p	Adjusted/stratified HR (95% CI); p	Adjustment
Overall survival	60/17	17/4; 50.8 (36.3-63.8)	43/13; 57.2 (46.5-NR)	0.580	1.43 (0.40-5.05); 0.580	2.00 (0.55-7.29); 0.293	Adjusted for age (per 10 years)
Disease-free survival	44/16	13/5; 22.8 (21.8-29.9)	31/11; 45.5 (24.8-NR)	0.175	0.46 (0.15-1.45); 0.185	0.87 (0.28-2.67); 0.808	Stratified by histological grade
Progression-free survival	16/14	4/4; 11.9 (9.1-22.8)	12/10; 14.4 (5.3-22.4)	0.503	0.67 (0.20-2.20); 0.505	Not fitted	Not fitted because of the small cohort

For OS, the adjusted model included age per 10-year increase and PD-L1 status. For DFS, the PD-L1 HR was estimated in a Cox model stratified by histological grade because the grade effect was non-proportional. The PFS model was unadjusted because of the small cohort. NR, not reached.

**Figure 4:**
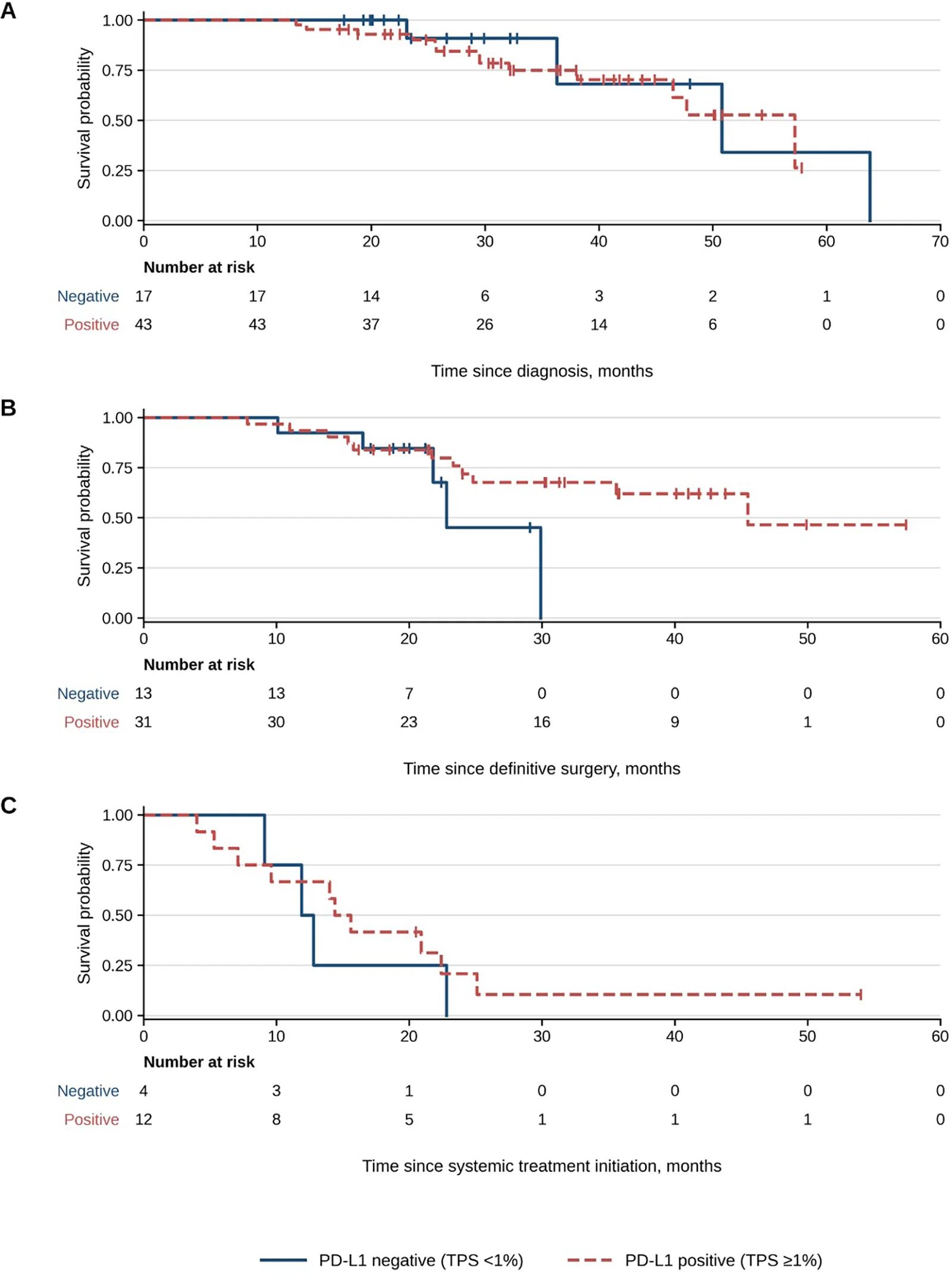
Kaplan-Meier curves according to binary PD-L1 status. **(A)** Overall survival in all 60 patients. **(B)** Disease-free survival in 44 patients rendered disease-free after definitive treatment. **(C)** Progression-free survival in 16 patients with baseline metastatic or residual disease. Tick marks indicate censoring, and numbers at risk are shown below each panel. The corresponding curves for the three-level PD-L1 classification are shown in **Supplementary Figure S1**.

**Figure 5:**
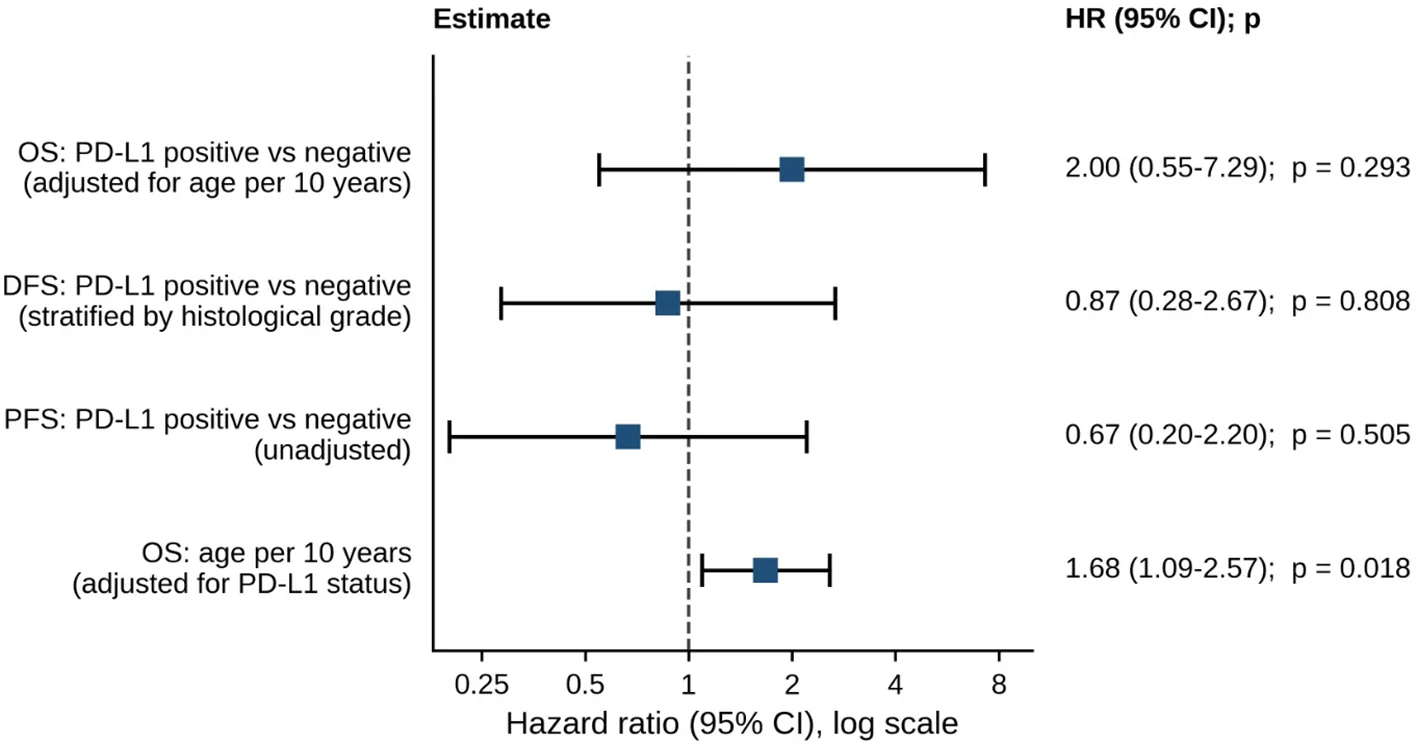
Exploratory Cox proportional-hazards estimates. Hazard ratios and 95% confidence intervals are shown on a logarithmic scale. The OS PD-L1 estimate was adjusted for age per 10 years; the DFS estimate was stratified by histological grade; the PFS estimate was unadjusted. Squares mark the point estimate and horizontal bars the 95% confidence interval; the dashed vertical line marks a hazard ratio of 1.

### Disease-free and progression-free survival

The DFS cohort comprised 44 patients who were disease-free after definitive treatment. Sixteen DFS events occurred: 14 recurrences (nine distant, three locoregional, and two involving both sites) and two deaths without documented recurrence. The median follow-up was 31.3 months, and the median DFS was 45.5 months. PD-L1 status was not associated with DFS (log-rank p=0.175; univariable HR 0.46, 95% CI 0.15-1.45; p=0.185). Because grade did not satisfy the proportional hazards assumption, a grade-stratified Cox model was used; the PD-L1 estimate remained null (HR 0.87, 95% CI 0.28-2.67; p=0.808). Distant recurrence occurred in 4/13 PD-L1-negative and 7/31 PD-L1-positive patients and did not differ according to PD-L1 status (Fisher’s exact p=0.706).

The PFS cohort comprised 16 patients, of whom 14 experienced progression or death. Median PFS was 14.0 months. There was no evidence of a difference by PD-L1 status (log-rank p=0.503; HR 0.67, 95% CI 0.20-2.20; p=0.505). MSI-H/dMMR status was not associated with OS (HR 1.30, 95% CI 0.45-3.78; p=0.624) or DFS (HR 1.85, 95% CI 0.64-5.36; p=0.256), although these comparisons were underpowered.

### Sensitivity analyses using the three-level PD-L1 classification

Because the ≥1% threshold combines weakly and strongly stained tumors, every analysis was repeated using a three-level classification. Across the same family of 10 baseline variables, MSI/MMR status (p=0.0017; q=0.014), sex (p=0.0028; q=0.014), and histological subtype (p=0.0115; q=0.038) were associated with the three-level category, whereas no variable was associated with the binary status. The MSI/MMR and subtype associations were monotonic across the ordered tiers (linear-by-linear trend p=0.0012 and p=0.011): MSI-H/dMMR accounted for 5.9%, 12.9%, and 58.3% of the negative, low-positive, and high-positive tiers, respectively, and mucinous adenocarcinoma accounted for 5.9%, 6.5%, and 41.7%, respectively. The association with sex was not monotonic (trend p=0.660) and was driven by a single female patient in the low-positive tier (1 of 31) against five of 17 negative and five of 12 high-positive tumors. With the PD-L1 category entered as an ordinal term, each one-tier increase was associated with MSI-H/dMMR status (odds ratio 6.19, 95% CI 2.06-23.25; p<0.001), and highly positive tumors were enriched for MSI-H/dMMR relative to all other tumors (odds ratio 12.04, 95% CI 2.88-57.50; p<0.001). The corresponding estimate for binary positivity was weaker and did not reach significance (odds ratio 5.50, 95% CI 0.94-105.01; p=0.060), consistent with the exact test for that comparison (p=0.151). Because several cells in these models were sparse, their confidence intervals were profile-likelihood intervals, and their p-values were likelihood ratio tests. No survival signal emerged at any tier: global log-rank p-values were 0.770 for OS, 0.245 for DFS, and 0.668 for PFS, and neither the ordinal nor the indicator parameterization was associated with any endpoint, with or without adjustment for age or stratification by grade (**Supplementary Tables S2**–**S4**; **Supplementary Figure S1**). Individual patient data for all 60 patients are provided in **Supplementary Table S1**.

## Discussion

### Principal findings

Using the ZR3 clone and a TPS ≥1% threshold, tumor-cell PD-L1 positivity was recorded in 71.7% of tumors, which is higher than most CRC tumor-cell TPS series and best interpreted as a property of the assay and threshold rather than a transferable prevalence estimate. Consistent with this, no baseline clinicopathological characteristic was associated with binary PD-L1 status after exact testing and false-discovery-rate correction, and across endpoint-specific populations, PD-L1 status was not associated with overall (OS), disease-free (DFS), or progression-free survival (PFS). The only signal that tracked established biology was the enrichment of MSI-H/dMMR tumors within the high-positive tier; MSI/MMR status was unrelated to the simpler positive-versus-negative classification, indicating that the ≥1% dichotomy grouped biologically dissimilar tumors (35, 40–42). Repeating every analysis across the three-level classification supported this reading: the association with MSI/MMR status strengthened and was monotonic across tiers, whereas no endpoint showed a survival difference at any tier; therefore, the dichotomy masked a biological gradient rather than a prognostic effect (**Supplementary Tables S2**–**S4**; **Supplementary Figure S1**). Overall, ZR3-based tumor-cell TPS did not behave as an independent clinicopathological or prognostic marker in this cohort, and any biological signal was confined to high rather than low staining.

### PD-L1 prevalence and clinicopathological associations

The 71.7% positivity exceeds most tumor-cell TPS series in CRC, in which tumor-cell positivity at ≥1% is usually substantially lower (21, 22, 29, 31, 32, 43), and several assay-related factors are most likely to contribute. PD-L1 classification is strongly influenced by the antibody clone, platform, preanalytical handling, evaluated compartment, scoring algorithm, and threshold; therefore, the same tumor may be categorized differently by different methods (19, 20). The TPS metric was developed for tumor-cell scoring in non-small-cell lung cancer rather than CRC, and the ZR3 clone is a laboratory-developed reagent that has not been validated as a CRC companion diagnostic. Because contemporaneous run sheets were not retained, retrieval conditions, incubation times, and lot numbers could not be reconstructed, and pre-consensus scores were unavailable for quantifying interobserver agreement. The distribution of positive cases is itself informative: 31 of the 43 positive tumors were low-positive and only 12 were high-positive; thus, the ≥1% cut-off captured a large group of weakly stained tumors whose biology differed from that of strongly stained tumors, as confirmed by the three-level analyses. Therefore, the observed positivity should be regarded as specific to the ZR3 clone, tumor-cell TPS, and chosen thresholds and as potentially inflated by clone- and threshold-related overcall rather than as a transferable prevalence estimate. Consistent with this, guidance emphasizes clone-, platform-, and scoring-specific interpretation (27, 28), and clone- and scoring-dependent variation is documented directly in CRC (30, 32, 44).

Regional and South Asian series illustrate this heterogeneity: PD-L1 expression has varied with clinicopathological features in Middle Eastern cohorts (45, 46) and has been linked to poor differentiation, tumor-infiltrating lymphocytes, and higher T or N categories in South Asian studies (23, 24), while the earlier Pakistani study by Ahmad et al. examined its relationship with tumor stage and the immune microenvironment (25). In the present cohort, binary PD-L1 positivity was not independently related to grade, age, site, stage, nodal status, or vascular or perineural invasion after multiplicity corrections. This absence does not contradict earlier reports; differences in case selection, stage distribution, antibody, sample size, and analysis all contribute, and nominal associations in small studies often do not survive correction. With only 17 PD-L1-negative tumors, the power to detect modest associations was limited; therefore, the null primary result reflects the absence of evidence under these assay and sample size conditions rather than evidence of no association.

### Relationship with MSI/MMR status

The enrichment of MSI-H/dMMR tumors in the high-positive PD-L1 category is biologically plausible: mismatch repair deficiency raises somatic mutational and neoantigen burden, promoting lymphocytic infiltration and interferon-mediated checkpoint upregulation, and molecular-pathological studies have linked tumor-cell PD-L1 to activated T-cell responses, MSI, BRAF mutation, medullary morphology, and cytotoxic tumor-infiltrating lymphocytes (41, 47). However, this relationship is complex. PD-L1-positive MSI tumors comprise heterogeneous immune subsets rather than uniform categories (42). Associations vary by evaluated compartment and immune context (48, 49), and tumor-cell TPS captures only one component of a broader immune response that also involves immune cell PD-L1 (50).

In this cohort, MSI/MMR status was associated with the three-level PD-L1 classification but not with binary positivity, indicating that collapsing low- and high-positive tumors obscures biologically meaningful variation; this finding reflects the enrichment of high-level PD-L1 in MSI-H/dMMR tumors rather than the equivalence of all PD-L1-positive tumors with MSI-H/dMMR. The higher frequency of MSI-H/dMMR in right-sided tumors was consistent with established CRC biology. The complete concordance between PCR-based MSI testing and four-protein MMR immunohistochemistry should be interpreted as internal consistency rather than proof of universal interchangeability, since discordance can arise from technical factors, retained antigenicity of non-functional proteins, or uncommon alterations. Notably, not all studies show a direct MSI–PD-L1 association; Noepel-Duennebacke et al. reported a high immune score in MSI metastatic CRC without increased PD-L1 expression or survival (51), reinforcing assay- and context-specific interpretations.

As this study used both methods, it is worth setting out what each contributes. MMR immunohistochemistry and PCR-based MSI testing interrogate different levels of the same defect and are therefore complementary, rather than interchangeable. Immunohistochemistry is inexpensive, available in any histopathology laboratory, requires no molecular infrastructure, can be performed on a single section, and identifies which protein is lost, which in turn directs MLH1 promoter methylation or BRAF testing and germline referral. For these reasons, it is recommended as a reasonable first-line assay in most settings, including resource-limited ones (35, 39). Its weaknesses are pre-analytical and interpretive: variable fixation and antigen retrieval, edge and crush artifact, weak or absent internal controls, heterogeneous or patchy staining, and subjective assessment of equivocal nuclei can all mislead, and a missense mutation may leave a catalytically inactive protein that is still antigenically intact, so that staining is retained in a genuinely deficient tumor (38, 52, 53). In contrast, PCR-based fragment analysis measures the functional consequences of mismatch repair loss and is independent of antigen preservation. The pentaplex mononucleotide panel is quasimonomorphic and permits tumor-only testing (33, 34), and the marker-count criteria for MSI-H, MSI-L, and MSS make the readout relatively objective and reproducible between laboratories (36, 37). Its constraints are equally real: it requires molecular infrastructure and trained staff, adequate DNA quantity and quality from archival blocks, and a sufficient neoplastic cell fraction, since a low tumor fraction dilutes unstable alleles and can generate false-negative calls; sensitivity is lower for MSH6-deficient tumors, in which instability may involve fewer markers, and the assay does not indicate which protein is affected (39, 54). Reported concordance between the two methods in colorectal cancer is high but not complete, and the residual discordance tracks these complementary failure modes (52, 54, 55). Current guidance therefore treats the two as mutually confirmatory, recommending the second method (or next-generation sequencing) to resolve equivocal, discordant, or clinically unexpected results rather than designating either as a universal standard (35, 38, 39). In the present cohort, the two assays agreed in all 60 cases, which is consistent with the literature; however, given the sample size and the absence of sequencing confirmation, that agreement should be read as internal consistency rather than as evidence of interchangeability.

### Survival interpretation

Because survival was a prespecified exploratory endpoint, these analyses should be interpreted cautiously. PD-L1 status was not associated with OS, DFS, or PFS, consistent with the inconsistent literature in which meta-analyses report adverse associations in selected analyses (21, 22); however, the magnitude and direction vary by ethnicity, stage, scoring method, cutoff, and cellular localization (43), and individual cohorts differ according to compartment and analytical approach (56–58). The apparent sub-unity DFS hazard ratio moved toward the null after grade stratification and reflected imprecision from a few events rather than a protective effect. The PFS comparison, with 16 patients and four PD-L1-negative cases, was less precise; therefore, non-significance must not be read as equivalence. Age per 10-year increase was associated with mortality independent of PD-L1 status; however, with only 17 deaths and no external validation, this is a hypothesis-generating rather than a validated prognostic model.

### Clinical implications

These findings do not support the addition of ZR3-based tumor cell PD-L1 TPS to CRC treatment selection algorithms. The results of this laboratory-developed assay are not interchangeable with those of approved PD-L1 platforms and should not be used to independently select or exclude patients from immune checkpoint therapy. MSI/MMR status remains the clinically actionable immunotherapy biomarker in CRC, with pembrolizumab and nivolumab showing meaningful activity in MSI-H/dMMR disease (14–17), whereas PD-L1 TPS is not an established treatment-selection test (18). The concordance between PCR-based MSI testing and four-protein MMR immunohistochemistry supports MMR immunohistochemistry as a pragmatic initial approach where molecular infrastructure is limited, while equivocal staining, absent internal controls, or unexpected loss patterns may require molecular resolution per colorectal biomarker guidance (38).

### Strengths and limitations

The strengths of this study include the combined evaluation of tumor-cell PD-L1, PCR-based MSI, four-protein MMR immunohistochemistry, and longitudinal outcomes in one cohort, and an analysis using exact tests for sparse tables, effect sizes, false-discovery-rate correction, endpoint-specific survival populations, and proportional-hazards checking. Separating the OS, DFS, and PFS populations prevented the misclassification of persistent or metastatic disease as disease-free. Repeating every analysis across the three-level PD-L1 classification and releasing the individual patient data as **Supplementary Table S1** allowed readers to judge the effect of the threshold for themselves.

The limitations of this study warrant emphasis. This was a single-center study of 60 patients with male and stage III–IV predominance, which limited the precision and generalizability of the results. The ZR3 assay is laboratory-developed and unvalidated as a CRC companion diagnostic, and documented clone- and scoring-related variability (19, 20, 30–32) means that the reported prevalence should not be generalized to other platforms. The ≥1% cut-off itself is not validated in CRC; it was transferred from non-small-cell lung cancer, where it was established for a different assay and a different clinical decision, and no CRC-specific threshold has been defined. Survival analyses were exploratory and underpowered, with heterogeneous treatments and possible residual confounding by comorbidity, performance status, and subsequent therapy. Potentially informative molecular variables, such as BRAF and RAS mutations, CpG island methylator phenotype, tumor mutational burden, and other immune markers, were unavailable. A single representative block per patient may not capture the intratumoral heterogeneity. Pre-consensus scores were not retained, precluding interobserver agreement estimation. The microscopy composites lack calibrated scale bars. Another limitation concerns the form in which the score was captured. The numerical tumor proportion score was not documented. At the time of scoring, each case was assigned directly to one of the three prespecified categories, and the underlying percentage was never entered into the database. Therefore, the tumor proportion score cannot be modeled as a continuous variable, no dose-response relationship can be estimated across the full range of staining, and a “no-call” band immediately above the 1% cut-off cannot be examined even retrospectively, because those values do not exist rather than merely being unavailable to us. The three-level analyses reported here, including ordinal parameterization, are the closest available approximations. Studies using this assay should record the exact percentage for every case, since the threshold effects demonstrated here can only be characterized properly on a continuous scale. The associations of the three-level category with sex and histological subtype arose in a family of 10 tests within 60 patients, resting on very small cells, and are hypothesis-generating only; the non-monotonic pattern for sex, in particular, is most consistent with sampling variation. As a retrospective study, it remains susceptible to selection bias and unmeasured confounding, and all time-to-event estimates require external validation.

### Future research

Future studies should use adequately powered multicenter prospective Pakistani cohorts with validated and clearly documented PD-L1 platforms, standardized preanalytical procedures, prespecified scoring, and blinded reproducibility assessment, and should evaluate multiple regions or whole-slide digital scoring to quantify heterogeneity. PD-L1 has been best studied as one element of an integrated immune and molecular profile alongside MSI/MMR, BRAF, RAS, tumor mutational burden, tumor and immune-cell PD-L1, tumor-infiltrating lymphocytes, and digital spatial pathology (41, 42, 47–50) to determine whether a composite adds prognostic value. Survival studies should prespecify endpoint-specific populations and include adequate numbers of events.

## Conclusion

Tumor-cell PD-L1 expression was frequently recorded using the ZR3 assay in this Pakistani colorectal carcinoma cohort, and MSI-H/dMMR tumors were disproportionately represented in the high-positive TPS category. However, binary PD-L1 status was not associated with baseline clinicopathological characteristics or overall, disease-free, or progression-free survival. These findings indicate that ZR3-based tumor-cell PD-L1 TPS should be interpreted as an assay-specific research measure and should not be used independently for prognostic stratification or immunotherapy selection in CRC patients. MSI/MMR testing remains a clinically actionable biomarker for molecular triage. Larger multicenter studies using validated PD-L1 platforms, standardized scoring methods, and adequately powered outcome analyses are required to determine whether PD-L1 adds prognostic or predictive value beyond that of established CRC biomarkers.

## Data Availability

The datasets presented in this study can be found in the article and its **Supplementary Material**. Individual patient-level data for all 60 patients are provided in **Supplementary Table S1**. Further inquiries can be directed to the corresponding author on reasonable request.
