# Supplementary material for: Tumor-cell PD-L1 expression, mismatch repair status, and survival outcomes in colorectal carcinoma: a retrospective Pakistani cohort study
Source: Front Oncol. 2026 Sep 14;16:1939073. doi: 10.3389/fonc.2026.1939073 (PMC13616628; doi:10.3389/fonc.2026.1939073)
Supplement: Supplementary file 1 [file DataSheet1.docx]

**Supplementary Material**

*Tumor-cell PD-L1 expression, mismatch repair status, and survival outcomes in colorectal carcinoma: a retrospective Pakistani cohort study*

**Supplementary Table S1. Individual patient data for all 60 cases.**

| **Case** | **Age** | **Sex** | **Site** | **Subtype** | **Grade** | **Stage** | **Node** | **VI** | **PNI** | **PD-L1 tier** | **PD-L1 ≥1%** | **MSI/MMR** | **MMR loss** | **Intent** | **Treatment** | **OS mo** | **OS ev** | **DFS mo** | **DFS ev** | **PFS mo** | **PFS ev** |
| --- | --- | --- | --- | --- | --- | --- | --- | --- | --- | --- | --- | --- | --- | --- | --- | --- | --- | --- | --- | --- | --- |
| P01 | 31 | F | R | AC | WD | III | N1-2 | – | – | High | + | MSS | – | Cur | C | 32.5 | 0 | 31.3 | 0 |  |  |
| P02 | 34 | M | L | AC | MD | II | N0 | – | – | High | + | MSI-H | MLH1/PMS2 | Cur | C | 31.4 | 0 | 30.2 | 0 |  |  |
| P03 | 36 | F | L | AC | PD | III | N1-2 | + | – | High | + | MSS | – | Cur | C | 38.4 | 0 | 24.8 | 1 |  |  |
| P04 | 38 | M | L | MAC | MD | III | N1-2 | – | – | High | + | MSS | – | Cur | C | 30.7 | 0 | 30.3 | 0 |  |  |
| P05 | 39 | F | R | AC | WD | III | N1-2 | – | – | High | + | MSI-H | MLH1/PMS2 | Cur | C | 36.6 | 0 | 35.8 | 0 |  |  |
| P06 | 43 | M | R | MAC | PD | III | N1-2 | + | – | High | + | MSI-H | MSH6 | Cur | C | 28.6 | 0 | 7.8 | 1 |  |  |
| P07 | 49 | F | L | AC | WD | IV | N0 | + | + | High | + | MSI-H | MSH2/MSH6 | Pal | C+IO | 23.7 | 1 |  |  | 4.0 | 1 |
| P08 | 51 | M | L | MAC | MD | IV | N0 | + | – | High | + | MSS | – | Cur | C | 54.3 | 0 |  |  | 54.0 | 0 |
| P09 | 52 | F | R | MAC | PD | III | N1-2 | + | – | High | + | MSI-H | MLH1/PMS2 | Cur | C | 38.0 | 0 | 24.0 | 1 |  |  |
| P10 | 53 | M | R | MAC | PD | III | N1-2 | + | – | High | + | MSI-H | MSH6 | Cur | C | 50.1 | 0 | 15.4 | 1 |  |  |
| P11 | 70 | M | L | AC | MD | IV | N1-2 | + | – | High | + | MSI-H | MSH2/MSH6 | Pal | C+IO | 29.5 | 1 |  |  | 20.9 | 1 |
| P12 | 80 | M | L | AC | MD | IV | N1-2 | + | + | High | + | MSS | – | Pal | C | 32.1 | 1 |  |  | 15.6 | 1 |
| P13 | 27 | M | R | AC | PD | II | N0 | – | – | Low | + | MSS | – | Cur | C | 30.3 | 0 | 23.3 | 1 |  |  |
| P14 | 27 | M | L | AC | MD | IV | N0 | + | – | Low | + | MSS | – | Cur | C+TT | 21.2 | 0 |  |  | 20.5 | 0 |
| P15 | 28 | M | R | AC | WD | III | N1-2 | – | – | Low | + | MSS | – | Cur | C | 22.5 | 0 | 21.5 | 0 |  |  |
| P16 | 30 | M | L | AC | WD | III | N1-2 | – | – | Low | + | MSS | – | Cur | C | 18.8 | 0 | 18.5 | 0 |  |  |
| P17 | 36 | M | R | AC | PD | III | N1-2 | + | – | Low | + | MSS | – | Cur | C | 30.3 | 0 | 11.0 | 1 |  |  |
| P18 | 37 | M | L | AC | WD | II | N0 | – | – | Low | + | MSS | – | Cur | C | 40.4 | 0 | 40.1 | 0 |  |  |
| P19 | 39 | M | L | MAC | MD | III | N1-2 | – | – | Low | + | MSS | – | Cur | C | 43.8 | 0 | 42.7 | 0 |  |  |
| P20 | 41 | M | R | AC | MD | III | N1-2 | – | – | Low | + | MSS | – | Cur | C | 42.6 | 0 | 41.8 | 0 |  |  |
| P21 | 41 | M | L | AC | MD | III | N1-2 | – | – | Low | + | MSS | – | Cur | C | 36.3 | 0 | 35.7 | 0 |  |  |
| P22 | 44 | M | R | AC | MD | III | N1-2 | – | – | Low | + | MSS | – | Cur | C | 32.2 | 0 | 31.7 | 0 |  |  |
| P23 | 49 | M | L | MAC | MD | II | N0 | – | – | Low | + | MSS | – | Cur | C | 24.8 | 0 | 24.0 | 0 |  |  |
| P24 | 53 | M | L | AC | PD | III | N1-2 | + | – | Low | + | MSS | – | Cur | C+RT | 26.4 | 0 | 21.7 | 1 |  |  |
| P25 | 56 | M | L | AC | PD | IV | N1-2 | + | + | Low | + | MSS | – | Pal | C+TT | 47.7 | 1 |  |  | 22.4 | 1 |
| P26 | 59 | M | R | AC | MD | IV | N0 | + | + | Low | + | MSS | – | Pal | C+TT | 25.6 | 1 |  |  | 7.1 | 1 |
| P27 | 62 | M | L | AC | MD | III | N1-2 | – | – | Low | + | MSS | – | Cur | C+RT | 57.8 | 0 | 57.4 | 0 |  |  |
| P28 | 62 | M | R | AC | PD | III | N1-2 | + | – | Low | + | MSI-H | MSH2/MSH6 | Cur | C | 41.3 | 0 | 15.8 | 1 |  |  |
| P29 | 62 | F | L | AC | WD | III | N1-2 | – | – | Low | + | MSS | – | Cur | C | 50.8 | 0 | 49.9 | 0 |  |  |
| P30 | 63 | M | R | AC | MD | II | N0 | – | – | Low | + | MSS | – | Cur | C | 44.9 | 0 | 43.8 | 0 |  |  |
| P31 | 64 | M | L | AC | MD | III | N1-2 | – | – | Low | + | MSS | – | Cur | C | 21.7 | 0 | 21.4 | 0 |  |  |
| P32 | 66 | M | L | AC | WD | IV | N1-2 | + | + | Low | + | MSS | – | Pal | C+TT | 29.5 | 1 |  |  | 14.4 | 1 |
| P33 | 67 | M | R | AC | PD | III | N1-2 | + | + | Low | + | MSS | – | Cur | C | 18.8 | 1 |  |  | 9.6 | 1 |
| P34 | 69 | M | L | AC | PD | IV | N0 | + | + | Low | + | MSS | – | Pal | C+TT | 25.7 | 1 |  |  | 14.0 | 1 |
| P35 | 71 | M | L | AC | MD | III | N1-2 | – | – | Low | + | MSS | – | Cur | C | 17.2 | 0 | 16.2 | 0 |  |  |
| P36 | 72 | M | L | AC | WD | III | N1-2 | – | – | Low | + | MSS | – | Cur | C | 50.2 | 0 | 49.9 | 0 |  |  |
| P37 | 73 | M | L | AC | MD | III | N1-2 | – | – | Low | + | MSS | – | Cur | C | 18.0 | 0 | 17.3 | 0 |  |  |
| P38 | 73 | M | R | AC | MD | IV | N1-2 | + | – | Low | + | MSS | – | Pal | C+TT | 13.4 | 1 |  |  | 5.3 | 1 |
| P39 | 73 | M | R | AC | MD | III | N1-2 | – | – | Low | + | MSI-H | MLH1/PMS2 | Cur | C | 41.8 | 0 | 41.0 | 0 |  |  |
| P40 | 74 | M | L | AC | WD | II | N0 | – | – | Low | + | MSS | – | Cur | C | 14.3 | 1 | 13.9 | 1 |  |  |
| P41 | 74 | M | R | AC | PD | IV | N0 | + | + | Low | + | MSI-H | MLH1/PMS2 | Pal | C+IO | 57.2 | 1 |  |  | 25.1 | 1 |
| P42 | 75 | M | L | AC | PD | III | N1-2 | + | – | Low | + | MSS | – | Cur | C | 38.1 | 1 | 35.6 | 1 |  |  |
| P43 | 76 | M | L | AC | PD | II | N0 | – | – | Low | + | MSI-H | PMS2 | Cur | C | 46.5 | 1 | 45.5 | 1 |  |  |
| P44 | 27 | M | L | MAC | PD | III | N1-2 | + | – | Neg | – | MSS | – | Cur | C | 48.0 | 0 | 16.5 | 1 |  |  |
| P45 | 28 | M | L | AC | PD | IV | N0 | + | + | Neg | – | MSS | – | Pal | C | 23.1 | 1 |  |  | 11.9 | 1 |
| P46 | 29 | M | L | AC | PD | II | N0 | – | – | Neg | – | MSS | – | Cur | C+RT | 17.6 | 0 | 17.1 | 0 |  |  |
| P47 | 51 | M | L | AC | PD | III | N1-2 | + | – | Neg | – | MSS | – | Cur | C+RT | 28.8 | 0 | 22.8 | 1 |  |  |
| P48 | 51 | F | R | AC | MD | III | N1-2 | – | – | Neg | – | MSS | – | Cur | C | 29.9 | 0 | 29.1 | 0 |  |  |
| P49 | 54 | M | L | AC | WD | II | N0 | – | – | Neg | – | MSS | – | Cur | C | 23.5 | 0 | 22.4 | 0 |  |  |
| P50 | 56 | M | L | AC | PD | III | N1-2 | + | – | Neg | – | MSS | – | Cur | C | 32.2 | 0 | 29.9 | 1 |  |  |
| P51 | 62 | F | L | AC | MD | II | N0 | – | – | Neg | – | MSS | – | Cur | C | 22.4 | 0 | 21.2 | 0 |  |  |
| P52 | 63 | M | R | AC | MD | II | N0 | – | – | Neg | – | MSS | – | Cur | C | 19.3 | 0 | 18.8 | 0 |  |  |
| P53 | 63 | M | L | AC | MD | III | N1-2 | – | – | Neg | – | MSS | – | Cur | C | 19.9 | 0 | 19.6 | 0 |  |  |
| P54 | 64 | M | L | AC | PD | II | N0 | – | – | Neg | – | MSS | – | Cur | C | 20.1 | 0 | 18.8 | 0 |  |  |
| P55 | 67 | F | L | AC | WD | II | N0 | – | – | Neg | – | MSS | – | Cur | C+RT | 21.1 | 0 | 20.0 | 0 |  |  |
| P56 | 68 | M | R | AC | PD | III | N1-2 | + | + | Neg | – | MSI-H | MLH1/PMS2 | Cur | C | 36.3 | 1 |  |  | 22.8 | 1 |
| P57 | 70 | F | L | AC | WD | III | N1-2 | + | – | Neg | – | MSS | – | Cur | C+RT | 26.6 | 0 | 10.1 | 1 |  |  |
| P58 | 71 | M | L | AC | PD | IV | N0 | + | + | Neg | – | MSS | – | Pal | C | 63.8 | 1 |  |  | 12.8 | 1 |
| P59 | 80 | M | L | AC | MD | IV | N1-2 | + | + | Neg | – | MSS | – | Pal | C+TT | 50.8 | 1 |  |  | 9.1 | 1 |
| P60 | 80 | F | L | AC | PD | II | N0 | + | – | Neg | – | MSS | – | Cur | C+RT | 32.8 | 0 | 21.8 | 1 |  |  |

*Cases are ordered by PD-L1 tumor proportion score (TPS) category and then by age; case identifiers are study-specific and cannot be traced to individual patients. PD-L1 is reported as the assigned TPS category: the numerical percentage was not recorded at the time of consensus scoring, so the ordered category is the finest-grained PD-L1 measurement available for this cohort. Event indicators (OS ev, DFS ev, PFS ev): 1 = event, 0 = censored. Blank time and event cells indicate that the patient did not contribute to that endpoint-specific analysis population (disease-free survival was restricted to the 44 patients rendered disease-free after definitive treatment; progression-free survival to the 16 patients with baseline metastatic or residual disease). The same data with unabbreviated labels are provided as a machine-readable spreadsheet.*

*Key to coded values. Sex: M, male; F, female. Site: L, left-sided; R, right-sided. Subtype: AC, adenocarcinoma; MAC, mucinous adenocarcinoma. Grade: WD, well differentiated; MD, moderately differentiated; PD, poorly differentiated. Node: N0, pN0; N1-2, pN1-pN2. VI, vascular invasion; PNI, perineural invasion (+, present; –, absent). PD-L1 tier: Neg, TPS <1%; Low, TPS 1-49%; High, TPS ≥50%. PD-L1 ≥1%: +, positive; –, negative. MSI/MMR: MSS, microsatellite stable / mismatch-repair proficient; MSI-H, microsatellite instability-high / mismatch-repair deficient. MMR loss: proteins with complete loss of tumor-cell nuclear staining (–, all four retained). Intent: Cur, curative; Pal, palliative. Treatment: C, chemotherapy; RT, radiotherapy; TT, targeted therapy; IO, immunotherapy. AJCC, American Joint Committee on Cancer; DFS, disease-free survival; mo, months; OS, overall survival; PFS, progression-free survival; TPS, tumor proportion score.*

**Supplementary Table S2. Baseline clinicopathological characteristics according to the three-level PD-L1 tumor proportion score category.**

| **Variable** | **Negative (n=17)** | **Low positive (n=31)** | **High positive (n=12)** | **p** | **q (BH)** | **Effect size** |
| --- | --- | --- | --- | --- | --- | --- |
| Age, years (Kruskal-Wallis) | 63.0 (51.0-68.0) | 62.0 (41.0-71.5) | 46.0 (37.5-52.2) | 0.2273 | 0.3901 | - |
| Sex | Female 5 (29.4%); Male 12 (70.6%) | Female 1 (3.2%); Male 30 (96.8%) | Female 5 (41.7%); Male 7 (58.3%) | 0.0028 | 0.0138 | Cramer's V = 0.418 |
| Histological subtype | Adenocarcinoma 16 (94.1%); Mucinous adenocarcinoma 1 (5.9%) | Adenocarcinoma 29 (93.5%); Mucinous adenocarcinoma 2 (6.5%) | Adenocarcinoma 7 (58.3%); Mucinous adenocarcinoma 5 (41.7%) | 0.0115 | 0.0382 | Cramer's V = 0.417 |
| Histological grade | Moderately differentiated 5 (29.4%); Poorly differentiated 9 (52.9%); Well differentiated 3 (17.6%) | Moderately differentiated 14 (45.2%); Poorly differentiated 10 (32.3%); Well differentiated 7 (22.6%) | Moderately differentiated 5 (41.7%); Poorly differentiated 4 (33.3%); Well differentiated 3 (25.0%) | 0.7170 | 0.7967 | Cramer's V = 0.136 |
| Tumor site | Left-sided 14 (82.4%); Right-sided 3 (17.6%) | Left-sided 19 (61.3%); Right-sided 12 (38.7%) | Left-sided 7 (58.3%); Right-sided 5 (41.7%) | 0.2553 | 0.3901 | Cramer's V = 0.211 |
| Pathological stage | II 7 (41.2%); III 7 (41.2%); IV 3 (17.6%) | II 6 (19.4%); III 18 (58.1%); IV 7 (22.6%) | II 1 (8.3%); III 7 (58.3%); IV 4 (33.3%) | 0.3162 | 0.3953 | Cramer's V = 0.206 |
| Lymph node status | pN0 9 (52.9%); pN1-pN2 8 (47.1%) | pN0 10 (32.3%); pN1-pN2 21 (67.7%) | pN0 3 (25.0%); pN1-pN2 9 (75.0%) | 0.2730 | 0.3901 | Cramer's V = 0.220 |
| Vascular invasion | Absent 8 (47.1%); Present 9 (52.9%) | Absent 19 (61.3%); Present 12 (38.7%) | Absent 4 (33.3%); Present 8 (66.7%) | 0.2484 | 0.3901 | Cramer's V = 0.220 |
| Perineural invasion | Absent 13 (76.5%); Present 4 (23.5%) | Absent 25 (80.6%); Present 6 (19.4%) | Absent 10 (83.3%); Present 2 (16.7%) | 0.9157 | 0.9157 | Cramer's V = 0.061 |
| MSI/MMR status | MSI-H / dMMR 1 (5.9%); MSS / pMMR 16 (94.1%) | MSI-H / dMMR 4 (12.9%); MSS / pMMR 27 (87.1%) | MSI-H / dMMR 7 (58.3%); MSS / pMMR 5 (41.7%) | 0.0017 | 0.0138 | Cramer's V = 0.485 |

*Values are n (%) unless otherwise stated; age is median (interquartile range). p-values are from the Kruskal-Wallis test for age and the Fisher-Freeman-Halton exact test for categorical variables. Benjamini-Hochberg (BH) q-values control the false discovery rate across the same family of 10 baseline variables used in the primary binary analysis. This three-level family is a sensitivity analysis and is secondary to the binary primary analysis reported in Table 1; it is interpreted as exploratory.*

**Supplementary Table S3. Time-to-event models using the three-level PD-L1 category.**

| **Endpoint** | **Model** | **Term** | **n/events** | **HR (95% CI)** | **p** |
| --- | --- | --- | --- | --- | --- |
| Overall survival | Univariable, ordinal tier | PD-L1 tier, per one-tier increase | 60/17 | 1.07 (0.52-2.23) | 0.852 |
| Overall survival | Univariable, tier indicators | Low positive vs negative | 60/17 | 1.54 (0.42-5.62) | 0.515 |
| Overall survival | Univariable, tier indicators | High positive vs negative | 60/17 | 1.14 (0.23-5.77) | 0.870 |
| Overall survival | Adjusted for age, ordinal tier | PD-L1 tier, per one-tier increase | 60/17 | 1.53 (0.69-3.41) | 0.295 |
| Overall survival | Adjusted for age, ordinal tier | Age, per 10 years | 60/17 | 1.70 (1.11-2.61) | 0.015 |
| Overall survival | Adjusted for age, tier indicators | Low positive vs negative | 60/17 | 1.94 (0.52-7.24) | 0.324 |
| Overall survival | Adjusted for age, tier indicators | High positive vs negative | 60/17 | 2.33 (0.42-12.76) | 0.331 |
| Overall survival | Adjusted for age, tier indicators | Age, per 10 years | 60/17 | 1.70 (1.10-2.62) | 0.017 |
| Disease-free survival | Univariable, ordinal tier | PD-L1 tier, per one-tier increase | 44/16 | 0.83 (0.36-1.89) | 0.657 |
| Disease-free survival | Univariable, tier indicators | Low positive vs negative | 44/16 | 0.37 (0.10-1.29) | 0.118 |
| Disease-free survival | Univariable, tier indicators | High positive vs negative | 44/16 | 0.74 (0.19-2.89) | 0.660 |
| Disease-free survival | Stratified by grade, ordinal tier | PD-L1 tier, per one-tier increase | 44/16 | 1.07 (0.51-2.22) | 0.860 |
| Progression-free survival | Univariable, ordinal tier | PD-L1 tier, per one-tier increase | 16/14 | 0.71 (0.33-1.51) | 0.372 |
| Progression-free survival | Univariable, tier indicators | Low positive vs negative | 16/14 | 0.77 (0.22-2.70) | 0.680 |
| Progression-free survival | Univariable, tier indicators | High positive vs negative | 16/14 | 0.50 (0.11-2.33) | 0.376 |

*Cox proportional-hazards models. "Ordinal tier" enters the PD-L1 category as a single ordered term scored 0 (negative), 1 (low positive), and 2 (high positive); "tier indicators" enter low-positive and high-positive as separate indicators with negative as the reference. Global log-rank p-values across the three tiers were 0.7697 for overall survival, 0.2451 for disease-free survival, and 0.6676 for progression-free survival. The proportional-hazards assumption was satisfied for all three ordinal models (Schoenfeld residuals). CI, confidence interval; HR, hazard ratio.*

**Supplementary Table S4. Logistic regression models for MSI-H/dMMR status according to PD-L1 classification.**

| **PD-L1 parameterization** | **Term** | **Odds ratio (95% CI)** | **p** |
| --- | --- | --- | --- |
| Binary PD-L1 (TPS >=1%) | PD-L1 positive vs negative | 5.50 (0.94-105.01) | 0.060 |
| Ordinal PD-L1 tier (0/1/2) | PD-L1 tier, per one-tier increase | 6.19 (2.06-23.25) | <0.001 |
| Tier indicators (reference: negative) | Low positive vs negative | 2.37 (0.32-48.55) | 0.427 |
| Tier indicators (reference: negative) | High positive vs reference | 22.40 (3.01-476.22) | 0.001 |
| High-positive versus all other tumors | High positive vs negative or low positive | 12.04 (2.88-57.50) | <0.001 |

*Outcome is MSI-H/dMMR versus MSS/pMMR (12 versus 48 tumors). Each PD-L1 parameterization is a separate model; the ordinal parameterization scores the PD-L1 category 0, 1, and 2 as in Supplementary Table S3. Because the cells are sparse — only one MSI-H/dMMR tumor was PD-L1 negative — Wald intervals and Wald p-values are unreliable in these models. Confidence intervals are therefore profile-likelihood intervals and p-values are likelihood-ratio tests. The intervals remain wide, and the estimates should be read as identifying the direction and location of the association rather than its precise magnitude. For the binary comparison the reference test remains Fisher's exact test (p=0.151, Tables 1 and 3), which is the more conservative and more appropriate analysis of that sparse 2 x 2 table. CI, confidence interval; dMMR, deficient mismatch repair; MSI-H, microsatellite instability-high; MSS, microsatellite stable; pMMR, proficient mismatch repair.*

**Supplementary Figure S1. Kaplan-Meier curves according to the three-level PD-L1 tumor proportion score category.**

(A) Overall survival in all 60 patients. (B) Disease-free survival in the 44 patients rendered disease-free after definitive treatment. (C) Progression-free survival in the 16 patients with baseline metastatic or residual disease. Curves compare PD-L1-negative (tumor proportion score [TPS] <1%), low-positive (TPS 1-49%), and high-positive (TPS >=50%) tumors. Tick marks indicate censored observations and the numbers at risk are shown beneath each panel. No endpoint showed a significant difference across the three tiers (global log-rank p = 0.7697, 0.2451, and 0.6676 for overall, disease-free, and progression-free survival, respectively).
